# Brain activation during non-habitual speech production: Revisiting the effects of simulated disfluencies in fluent speakers

**DOI:** 10.1371/journal.pone.0228452

**Published:** 2020-01-31

**Authors:** Catherine Theys, Silvia Kovacs, Ronald Peeters, Tracy R. Melzer, Astrid van Wieringen, Luc F. De Nil

**Affiliations:** 1 School of Psychology, Speech and Hearing, College of Science, University of Canterbury, Christchurch, New Zealand; 2 New Zealand Institute of Language, Brain and Behaviour, University of Canterbury, Christchurch, New Zealand; 3 Department of Radiology, University Hospitals Leuven, Leuven, Belgium; 4 New Zealand Brain Research Institute, Christchurch, New Zealand; 5 Department of Medicine, University of Otago, Christchurch, New Zealand; 6 Experimentele ORL, KULeuven, Leuven, Belgium; 7 Department of Speech-Language Pathology, University of Toronto, Toronto, Canada; The Hong Kong Polytechnic University, HONG KONG

## Abstract

Over the past decades, brain imaging studies in fluently speaking participants have greatly advanced our knowledge of the brain areas involved in speech production. In addition, complementary information has been provided by investigations of brain activation patterns associated with disordered speech. In the present study we specifically aimed to revisit and expand an earlier study by De Nil and colleagues, by investigating the effects of simulating disfluencies on the brain activation patterns of fluent speakers during overt and covert speech production. In contrast to the De Nil et al. study, the current findings show that the production of voluntary, self-generated disfluencies by fluent speakers resulted in increased recruitment and activation of brain areas involved in speech production. These areas show substantial overlap with the neural networks involved in motor sequence learning in general, and learning of speech production, in particular. The implications of these findings for the interpretation of brain imaging studies on disordered and non-habitual speech production are discussed.

## Introduction

Brain imaging studies of fluent speakers have greatly advanced our knowledge of the brain areas involved in speech production [1–5]. Combined, these studies have shown that speech production is supported by a network of brain areas that includes the pre- and postcentral gyri, posterior inferior frontal gyri, medial and lateral premotor cortex, anterior insula, superior temporal gyri, posterior planum temporale region, basal ganglia and cerebellum [4]. These findings have led to the formulation and refinement of theoretical network models of speech production such as the DIVA (Directions into Velocities of Articulators) Model [6–8] and the Integrated State Feedback Control Model of Speech Control [9].

In addition to studies on fluent speech production, investigations of brain activation patterns associated with disordered speech have provided complementary information on the neural correlates of speech production. However, interpretation of group differences in functional brain activation between control participants and those who experience speech disorders is often complicated because people with speech disorders often use spontaneous or treatment-induced speech modulations (e.g., speech rate changes) [10]. Therefore, activation patterns in people with speech disorders likely reflect not only the underlying dysfunction, but also any compensatory speech strategies or coping mechanisms used. Indeed, studies have shown significant interactions between observed brain activation patterns and speech task modulations, even in adults without speech disorders. In one such study, Riecker and colleagues [11] assessed the effects of speech rate changes on functional brain activation in 8 healthy controls. With this study, the authors aimed to increase our understanding of the neural basis of speech motor control and the neural mechanisms at play in different types of dysarthria. Spastic and ataxic dysarthria are associated with decreases in speech rate, while people with hypokinetic dysarthria may show increased speech rates. Speech rate changes are therefore often targeted in the treatment of dysarthric speech. The authors observed a linear change in BOLD response in speech-related brain regions including the supplementary motor area, the left anterior insula, bilateral thalamus, bilateral sensorimotor cortex, cerebellum and basal ganglia, when fluent-speaking adults were asked to speak at three different self-generated syllable rates. More recently, Marchina and colleagues [3] studied the effect of speech repetition rate on neural activation in 12 healthy controls to identify regions that can support recovery from speech disorders and to aid the development of adaptive treatment protocols for non-fluent aphasia, dysarthria, apraxia of speech and stuttering. They observed a significant linear increase in activation in the bilateral superior temporal areas with self-generated speech rate changes, suggesting that sensory feedback corresponds directly to task demands. Interestingly, bilateral activation changes in the speech motor regions were also identified, but these were less robust when a speaker was using a speech rate close to their habitual rate. They interpreted this as indicating that those close-to-habitual rate changes are likely highly practiced, and thus may require less additional regional motor support. In addition to the effects of speech rate changes in control participants, a number of studies have focused on the effects of fluency-inducing conditions on brain activation patterns in people who stutter. These studies, using fluency-inducing speech tasks such as choral speech, metronome-timed speech and altered auditory feedback, showed similar activation differences between people who stutter (PWS) and fluent speakers as identified during habitual speech. This includes overactivation of the vermal region of the cerebellum, supplementary motor area and insula, as well as decreased activation in the left-sided precentral gyrus [12–14], for a recent review, see [15]. At the same time, these tasks also resulted in increased activity in the bilateral superior temporal cortices in both PWS and fluent speakers. These findings contrast with the reduced activation in these areas typically seen during habitual speech production in PWS compared to their fluently speaking peers [16–18]. Based on the role of the superior temporal cortices in auditory feedback during speech production [1] and the findings on speech rate changes discussed above, it is unclear to what extent these changes may be associated with speech task modulations rather than increased fluency during these fluency-inducing conditions.

To dissociate the effects of task modulations from those related to increased fluency, De Nil and colleagues [19] investigated the effects of voluntary simulated disfluency (i.e., *decreased* fluency) on brain activation in PWS and fluent controls. Similar to previous studies comparing brain activation in PWS compared to fluent control participants [13, 14, 20], they reported that PWS showed less left superior temporal gyrus activation during habitual speech. Interestingly, a within-group comparison in PWS of simulated disfluency compared to habitual speech showed an increase in activation in the bilateral superior temporal, primary motor, premotor and inferior lateral prefrontal cortices, as well as in the left-sided insula and right-sided supramarginal gyrus during the simulated task. As this increase in activation implicated areas similar to those previously identified during habitual speech in PWS, the authors concluded that at least some of the functional overactivations reported previously when comparing stuttering and nonstuttering adults may have reflected between-group differences in the level of automaticity, effort and attention present during speech production. However, in contrast to the stuttering participants, no significant activation differences were observed for the within-group comparisons between the simulated disfluency and habitual speech tasks in nonstuttering participants in this study. This was somewhat surprising, because if the observed activation increases were indeed associated with the effort and attention required to produce atypical speech, similar patterns of increased activation were expected to occur in the control participants. This interpretation is further supported by the findings on speech rate changes and fluency-inducing conditions in fluent speakers discussed above [3, 11, 17, 18]. One of the potential explanations for this unexpected finding discussed in the De Nil et al. [19] paper was that activation changes in the fluent speakers may have been less pronounced and remained sub-threshold because of methodological reasons (use of a lower field strength (1.5T) scanner, sparse scanning, and use of auditory stimuli).

Because a thorough understanding of the observed neural activation patterns in fluent speakers is crucial for the correct interpretation of imaging findings of typical and disordered speech production, we report here on an fMRI study aimed at further investigating the effects of simulating disfluencies on the brain activation patterns of fluently speaking participants. Based on the results from De Nil et al. [19] in PWS, and the results from studies on non-habitual speech production in fluent speakers [3, 11, 17, 18], we hypothesized that asking speakers to simulate speech disfluencies would lead to increased demands placed on the neural network involved in speech production [1, 21], resulting in overactivation in the bilateral superior temporal gyri and other sensorimotor areas involved in speech production, even in fluently speaking individuals. To overcome some of the limitations of the previous study investigating simulated disfluency [19], we designed a paradigm using visual instead of auditory stimuli and used a scanner with higher field strength (3T) to increase signal detection. In addition, we included a pseudoword reading task because such words have been found to increase disfluency in PWS [22] and nonword repetition tasks resulted in differences in speech motor dynamics in PWS [23, 24]. As a result, we hypothesized that simulating disfluency during pseudoword versus word reading would further increase speech effort, resulting in an additional increase in activation in brain areas involved in speech production. In addition, participants were asked to produce speech overtly as well as covertly, since it has been shown that using overt compared to covert conditions leads to differences in brain responses [1, 25].

## Methods

### Participants

Eleven volunteers (5 females, 6 males) without a history of speech, language or neurological problems participated in the study. Their age ranged between 24–60 years, with an average of 40 years. All participants were right-handed, as assessed with the Dutch Handedness Inventory [26, 27]. The study was conducted with ethical approval of the University Hospitals Leuven and all participants provided written informed consent in accordance with the Declaration of Helsinki prior to their participation.

### Functional brain imaging

Magnetic resonance imaging data were acquired using a 3T MR system (INTERA, Philips Medical Systems, Best, The Netherlands) with an 8-channel phased-array head coil. For functional imaging, a T2*-weighted single shot gradient echo—echo planar imaging (GE-EPI) sequence was used with an echo time (TE) and repetition time (TR) of 33ms and 3000ms, respectively. The image acquisition matrix was 80×80, with a field of view (FOV) of 230×230mm^2^. A sensitivity encoding reduction factor (SENSE) of 2 was used in the anterior-posterior direction. Thirty-five contiguous transversal slices of 4mm thickness each were acquired with a flip angle of 90°, resulting in a voxel size of 2.9×2.9×4mm^3^.

Participants were scanned using a block design paradigm in which one run consisted of 9 epochs of 10 trials each. Epochs were presented in sets of three, with the first epoch consisting of 10 character strings (baseline condition), the second of 10 words and the third of 10 pseudowords. A new stimulus was randomly presented every 3 seconds, which resulted in a total duration of 30 seconds per epoch. Word stimuli were 88 high frequency Dutch nouns [28], including 40 one-syllable, 36 two-syllable (e.g., vlin-der [butterfly]) and 12 three-syllable words (e.g., ka-bou-ter [gnome]). Pseudowords and character strings were custom created. Pseudowords were constructed by replacing consonants and vocals in the high-frequency words by other graphemes, thereby retaining the original consonant-vocal structure of the word, resulting in non-existent but readable Dutch words (e.g., vlinder → klimder, kabouter → fimouter). Strings of characters were constructed by replacing each letter in a word by a pre-defined character (e.g., ?-{.# (= ).

The stimuli were presented visually and participants were instructed to either passively look at strings of non-sense characters (baseline condition) or to read the (pseudo)words according to one of the following instructions:

- read silently (covert habitual speech production);
- read out loud (overt habitual speech production);
- read silently while repeating the first letter of the word multiple times (covert simulated disfluency);
- read out loud while repeating the first letter of the word multiple times (overt simulated disfluency).

No instructions were given regarding the number of letter repetitions that were required, and repetitions were self-paced. Participants practiced all tasks prior to the scanning session. Each condition was presented twice in randomized order, which resulted in a total of 8 runs presented to each participant.

For anatomical reference an additional high-resolution 3D T1-weighted turbo field echo (TFE) sequence was used with a TE of 4.60ms and a TR of 9.7ms (acquired voxel size 0.98×0.98×1mm^3^, acquisition matrix 256×182 (reconstruction matrix 384×354), FOV 250×230mm^2^, reconstructed voxel size of 0.65×0.65×1mm^3^). A SENSE reduction factor of 1.4 in right-left direction and 1.6 in anterior-posterior direction was used. In total 230 contiguous coronal slices were acquired.

### Data analysis

CAT12 (r1278, http://www.neuro.uni-jena.de/cat/), a toolbox of SPM12 (v7219, http://www.fil.ion.ucl.ac.uk/spm/), running in Matlab 9.3 (R2017b), was used to process T1-weighted structural images. Images were bias corrected, spatially normalized via DARTEL (using the MNI-registered template provided within CAT12), modulated to compensate for the effect of spatial normalization, and classified into gray matter (GM), white matter (WM), and cerebrospinal fluid (CSF), all within the same generative model [29]. Using SPM12, the functional EPI images were realigned and the mean functional image was coregistered to the T1-weighted image. Next, all images were warped into MNI space using the MRI-derived deformation fields [30]. The normalized EPI images were spatially smoothed with a Gaussian kernel of 6 mm full-width half-maximum. For display purposes, a study-specific mean T1-weighted image was created by averaging all normalized T1-weighted images; all functional results were overlaid on this image.

First-level single subject analysis was performed by modeling the different stimuli (characters, words and pseudowords) using a boxcar function convolved with the hemodynamic response function using the general linear model. Motion parameters were added in the general linear model as covariates of non-interest for the statistical analysis. Individual statistical parametric maps were generated for words and pseudowords, and for overt and covert reading, for the following contrasts: ‘habitual speech production versus passively looking at characters’, and ‘simulated disfluency versus passively looking at characters’. These contrast images were subsequently entered in a second-level group analysis.

The second level group analysis was modelled as a 3-way ANOVA with the following factors: task (habitual speech production or simulated disfluency), mode (covert or overt), and stimulus (words or pseudowords). Next, t-tests were run to investigate the specific contrasts of interest. All statistical analyses were considered significant at an uncorrected voxel-level threshold of p < .001, with a cluster-level FWE corrected threshold of p < .05. Anatomical localization of the peak activations was determined using Automated Anatomical Labeling (AAL) and the Harvard-Oxford cortical and sub-cortical atlases in FSLeyes (https://fsl.fmrib.ox.ac.uk/fsl/fslwiki/FSLeyes). The processed data files are available from https://osf.io/zc9xk/.

## Results

Significant main results were found for task, mode and stimulus. In line with these findings and our hypotheses, t-tests were run for the following contrasts: ‘simulated disfluency > habitual speech’ (1), ‘overt speech > covert speech’ (2), and ‘pseudowords > words’ (3). A significant interaction was present for ‘task × mode’, but not for the ‘mode × stimulus’, ‘task × stimulus’ and ‘mode × task × stimulus’ interactions. Based on these findings, additional ‘covert simulated disfluency > covert habitual speech’ (4), and ‘overt simulated disfluency > overt habitual speech’ (5) comparisons were made.

### Habitual versus non-habitual speech production

**Fig 1:**
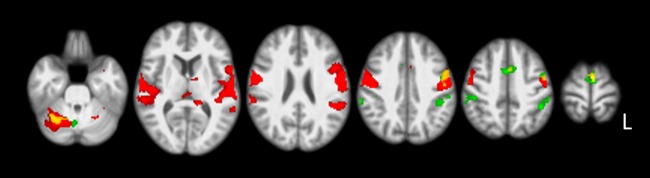
Simulated disfluencies compared to habitual speech. Figure showing ‘simulated disfluency > habitual speech’ (1) contrast results. For visualization purposes, these areas are overlaid on the ‘habitual speech > baseline’ contrast. Areas of red color indicate habitual speech versus baseline, and green areas indicate simulated disfluencies compared to habitual speech. Yellow indicates areas of overlap. Results were corrected for multiple comparisons using cluster-wise FWE (p<0.05), displayed on axial slices of the study specific normalized T1 template in MNI space (right of the image is left of brain). Slices displayed: z = -28, 9, 25, 41, 51, 73 mm.

For reference purposes, habitual speech production was compared to the baseline task of passively looking at characters. This resulted in large clusters of bilateral activation in the supplementary motor area (SMA), pre- and postcentral gyri, superior temporal gyri, bilateral thalamus and cerebellum. In addition, left-sided areas of significant activation were present in the fusiform gyrus, amygdala, hippocampus, and inferior frontal gyrus (pars triangularis). See Fig 1 (red color) and S1 Table. Comparison of the simulated disfluencies with the habitual speech task (‘simulated disfluency > habitual speech’ (1)), showed an increase in activation in clusters including the bilateral SMA, pre- and postcentral gyri and inferior partietal cortex. Additional activation was left-lateralized in the superior temporal gyrus and right-lateralized in the superior cerebellum (see Fig 1 and Table 1).

**Table 1:** Results on simulated disfluencies compared to habitual speech.

Anatomical region	Cluster		Peak	MNI coordinates		
	FWE corrected p-value	voxel extent	t-value	x	y	z
**L Supplementary motor area**	< 0.001	960	6.44	-5	5	57
**L Supplementary motor area**			6.07	-3	-6	69
**R Supplementary motor area**			4.88	6	7	67
**L Superior temporal gyrus**	0.014	186	5.47	-55	-12	3
**L Superior temporal pole**			4.76	-57	9	-8
**L Superior temporal gyrus**			4.02	-59	-2	-2
**R Cerebellum**	< 0.001	469	5.27	30	-56	-34
**R Cerebellum**			4.82	42	-60	-36
**R Cerebellum**			4.47	34	-56	-26
**L Inferior parietal lobule**	< 0.001	341	5.08	-45	-42	55
**L Inferior parietal lobule**			4.40	-57	-30	47
**L Inferior parietal lobule**			4.16	-47	-38	39
**R Postcentral gyrus**	0.004	245	4.8	44	-30	49
**R Inferior parietal lobule**			4.45	52	-30	57
**R Supramarginal gyrus**			4.27	62	-34	41
**L Precentral gyrus**	0.002	268	4.56	-51	5	47
**L Postcentral gyrus**			4.19	-49	-6	53
**L Postcentral gyrus**			4.16	-51	-2	39

Height threshold of p < 0.001 uncorrected, and cluster-based FWE-corrected p < 0.05 across the whole brain (cluster level threshold = 186 voxels). R = right; L = left.

**Fig 2:**
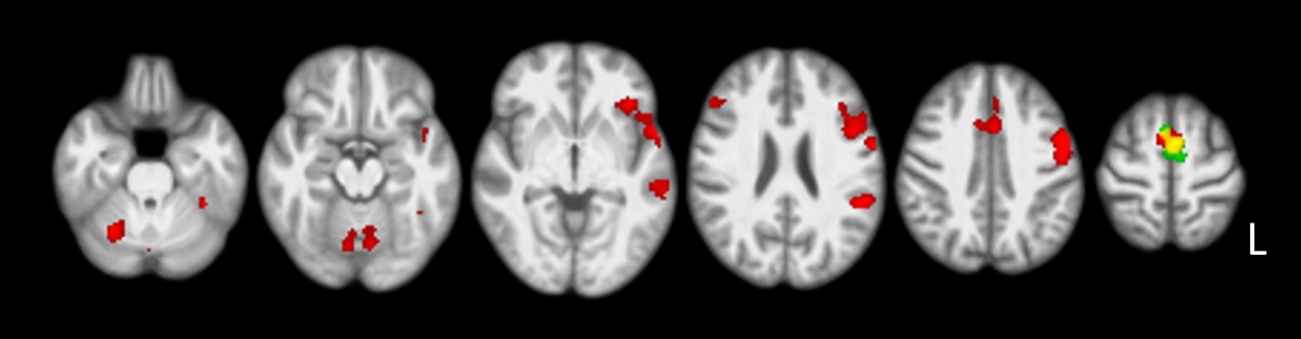
Covert simulated disfluencies compared to covert habitual speech. Figure showing ‘covert simulated disfluency > covert habitual speech’ (4) contrast results. For visualization purposes, these areas are overlaid on the ‘covert habitual speech > baseline’ contrast. Areas of red color indicate covert habitual versus baseline speech, and green areas indicate covert simulated disfluencies compared to covert habitual speech. Yellow indicates areas of overlap. Results were corrected for multiple comparisons using cluster-wise FWE (p<0.05), displayed on axial slices of the study specific normalized T1 template in MNI space (right of the image is left of brain). Slices displayed: z = -26, -16, -4, 25, 43, 63 mm.

The ‘task x mode’ interaction effect contrast showed one significant cluster of activation (FWE-corrected cluster p-value = 0.006, cluster extent = 186 voxels). This left-sided cluster had local maxima in Heschl’s gyrus [–55, –12, 3], the superior temporal gyrus [–59, –2, –2] and the temporal pole [–57, 7, –8]. Due to the ‘task × mode’ interaction effect, the effect of non-habitual speech production was assessed separately during covert and overt speech production. For reference purposes, the results on covert habitual speech production compared to the baseline are presented in S2 Table and included in Fig 2 (red areas). The comparison of covert simulated disfluency relative to covert habitual speech showed increased activation in the bilateral SMA (‘covert simulated disfluency > covert habitual speech’ (4), Fig 2 and Table 2).

**Table 2:** Results on covert simulated disfluencies compared to covert habitual speech.

Anatomical region	Cluster		Peak	MNI coordinates		
	FWE corrected p-value	voxel extent	t-value	x	y	z
**L Supplementary motor area**	< 0.001	675	6.04	-5	5	57
**L Supplementary motor area**			5.47	-3	-6	67
**R Supplementary motor area**			4.76	6	11	59

Height threshold of p < 0.001 uncorrected, and cluster-based FWE-corrected p < 0.05 across the whole brain (cluster level threshold = 675 voxels). R = right; L = left.

**Fig 3:**
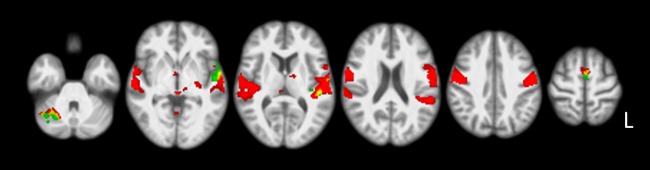
Overt simulated disfluencies compared to overt habitual speech. Figure showing ‘overt simulated disfluency > overt habitual speech’ (5) contrast results. For visualization purposes, these areas are overlaid on the ‘overt habitual speech > baseline’ contrast. Areas of red color indicate overt habitual versus baseline speech, and green areas indicate overt simulated disfluencies compared to overt habitual speech. Yellow indicates areas of overlap. Results were corrected for multiple comparisons using cluster-wise FWE (p<0.05), displayed on axial slices of the study specific normalized T1 template in MNI space (right of the image is left of brain). Slices displayed: z = -31, -2, 9, 21, 41, 63 mm.

During overt habitual speech production compared to the baseline, a large network of areas was activated. These are shown in S3 Table and Fig 3 (red color). The overt simulated disfluency task resulted in significant increased activations compared to overt habitual speech production. These increases were stronger than in the covert simulated disfluency task and included clusters in the bilateral SMA, the left-sided superior temporal gyrus and right-sided superior cerebellum (‘overt simulated disfluency > overt habitual speech’ (5), Fig 3 and Table 3).

**Table 3:** Results on overt simulated disfluencies compared to overt habitual speech.

Anatomical region	Cluster		Peak	MNI coordinates		
	FWE corrected p-value	voxel extent	t-value	x	y	z
**L Superior temporal gyrus**	< 0.001	616	6.23	-55	-12	3
**L Superior temporal gyrus**			5.36	-49	-28	7
**L Superior temporal gyrus**			4.88	-59	-2	-2
**R Cerebellum**	0.008	207	4.92	42	-60	-36
**R Cerebellum**			4.56	30	-56	-34
**R Cerebellum**			4.41	38	-70	-36
**L Supplementary motor area**	0.021	170	4.32	-5	3	59
**L Supplementary motor area**			4.25	-3	-4	71
**L Supplementary motor area**			3.65	-1	-6	61

Height threshold of p < 0.001 uncorrected, and cluster-based FWR-corrected p < 0.05 across the whole brain (cluster level threshold = 170 voxels). R = right; L = left.

### Overt versus covert speech production

Comparison of overt to covert speech production resulted in large clusters of bilateral activation, including the pre- and postcentral and superior temporal gyri, cerebellum, thalamus, amygdala and hippocampus (‘overt speech > covert speech’ (2), Table 4).

**Table 4:** Results on overt compared to covert speech.

Anatomical region	Cluster		Peak	MNI coordinates		
	FWE corrected p-value	voxel extent	t-value	x	y	z
**R Postcentral gyrus**	< 0.001	3531	16.7	56	-10	35
**R Postcentral gyrus**			14.77	64	-2	25
**R Superior temporal gyrus**			14.08	46	-12	3
**L Postcentral gyrus**	< 0.001	3166	14.71	-55	-12	31
**L Superior temporal gyrus**			13.82	-49	-26	7
**L Superior temporal gyrus**			12.76	-57	-12	3
**R Cerebellum**	< 0.001	2484	11.83	16	-62	-18
**L Cerebellum**			10.15	-15	-60	-20
**R Cerebellum**			6.62	32	-60	-28
**L Hippocampus**	0.004	241	7.20	-15	-6	-14
**L Amygdala**			6.04	-23	1	-12
**R Orbital frontal gyrus**			4.83	-17	15	-16
**R Hippocampus**	0.010	200	6.88	14	-6	-14
**R Amygdala**			6.64	28	1	-12
**L Thalamus**	< 0.001	556	6.09	-11	-8	13
**L Thalamus**			5.56	-9	-16	19
**L Brainstem**			5.26	2	-30	1
**R Thalamus**	0.007	218	5.82	16	-16	15

Height threshold of p < 0.001 uncorrected, and cluster-based FWE-corrected p < 0.05 across the whole brain (cluster level threshold = 200 voxels). R = right; L = left.

### Pseudoword versus word production

Pseudoword compared to word production (‘pseudowords > words’ (3)) resulted in one right-sided cluster in the inferior-medial occipital lobe (FWE-corrected cluster p-value = 0.044, cluster extent = 140 voxels), with local maxima at [34, –82, 11] and [38, –86, –2].

## Discussion

Speech task modulations, aimed at altering a person’s habitual speech pattern, give us an insight into the neural functioning of the speech network in healthy control speakers. Such task modulations are also important tools used in the assessment and treatment of speech disorders such as dysarthria, apraxia of speech and stuttering. Brain imaging studies focusing on both fluent and disordered speech have shown that non-habitual task modulations resulted in increased activation in speech-related areas [3, 11, 17, 18]. This effect was also seen in PWS when they were asked to speak in a dysfluent manner (i.e., simulate disfluencies), but no difference in activation was present in their fluent control group [19]. Therefore, the present study aimed specifically to revisit and expand the De Nil et al [19] study, by investigating the effects of simulating disfluencies on the brain activation patterns of fluent speakers during overt and covert speech production. In contrast to that study, the current findings showed that the production of voluntary, self-generated disfluencies by fluent speakers did result in increased recruitment and activation of brain areas involved in speech production. In addition, the non-habitual compared to the habitual speech task demands interacted with whether speech was produced overtly or covertly. Main effects of overt compared to covert speech production and the use of pseudowords compared to words were also identified.

### Habitual versus non-habitual speech production

When habitual speech production was compared to the baseline condition across both speaking modes (covert and overt, see Fig 1 and S1 Table), bilateral increases in activation were present in well-established speech-related areas [1, 4], with clusters including the supplementary motor area, precentral, postcentral and superior temporal gyrus, cerebellum and thalamus. In addition, significant left-sided activation was present in the left inferior frontal gyrus (pars triangularis), hippocampus, amygdala and fusiform gyrus. The latter, known as the visual word form area, is responsible for visual word recognition [31], and activation could be anticipated during the visual word reading task used in this study [32].

Compared to habitual speech production, using a non-habitual speech pattern by simulating disfluencies resulted in increased SMA activation, independent of whether speech was produced overtly or covertly (see Fig 1 and Table 1). Given that participants were asked to voluntarily repeat the first letter of the word multiple times as part of uttering the word, this activation is consistent with the view that the SMA is involved in the planning and initiation of speech, providing a starting mechanism for speech production [11, 33, 34]. Because producing multiple repetitions of the first letter of a word prior to completing the rest of the word is a highly atypical speech pattern for fluent speakers, the observed increase may have resulted from greater effort, and additional needed resources, associated with the timing and repeated initiation of the upcoming verbal utterance, including the provision of a syllabic frame for the speech signal, functions typically attributed to the SMA [4, 35, 36]. This interpretation is supported by observations of an increase in effective connectivity between the pre-SMA and dorsal premotor cortex as a result of an increased load on sequencing of motor plans when novel (i.e., non-habitual) combinations of syllables needed to be produced [37].

In addition to increased SMA activation, analysis of the main effect of simulated disfluencies compared to habitual speech showed that extra neural resources associated with the production of novel motor sequences were recruited [38–41]. This suggests that producing a novel non-habitual motor sequence required additional control of ongoing movements and error correction, similar to the activations seen when participants are repeatedly producing a new motor task [38–41]. The cerebellum has an important role in this process. For instance, in a study focused on transfer learning of novel motor sequences, more successful learning was associated with increased activity in the superior cerebellum as well as in the left dorsal premotor cortex, extending into the pre-SMA [42]. These same areas also showed increased activation in the current task contrast. Furthermore, interactions between the cerebellum and primary motor area also appear to be crucial for motor sequence learning and are likely to influence the final representation of the sequence in the primary motor area [40].

Besides production of novel motor sequences in general, the findings of the current study concur with those specifically focusing on speech production. When producing speech, communication between areas involved in motor, auditory and somatosensory processing through feedback and feedforward mechanisms is crucial [6, 21]. The projections from premotor to primary motor cortex, as well as cerebellar projections are involved in learning and maintaining feedforward commands for the overt production of syllables according to the DIVA-model of speech production [6, 7]. Auditory and somatosensory information processed in the superior temporal and inferior parietal cortex plays an essential role in maintaining the speech feedback control system. Likewise, in the Dual-Stream Model of Speech Processing [21], connections through the Dorsal Auditory-Motor Stream have been regarded as important for speech development and producing new sequences, with the latter continuing to be important in adults. This dorsal stream was further detailed in the Integrated State Feedback Control Model of Speech Control [9], which suggests that the sensorimotor integration in speech relies on a neural network including the superior temporal sulcus and gyrus, posterior planum temporale, ventral and dorsolateral regions of the premotor cortex, and the cerebellum. Our current findings of non-habitual compared to habitual speech production map nicely onto these theoretical models. In addition, a study on increased sequence complexity during syllable production led to the same increases in SMA and parietal cortex bilaterally, while activation was strongly left-lateralized in the pre- and postcentral gyri and right-lateralized in the cerebellum during overt speech production [1]. Together, this shows that speaking in a non-habitual manner by simulating disfluencies increases the demands on the feedforward and feedback systems involved in speech production.

The production of non-habitual overt speech resulted in increased left-sided auditory activation. Although auditory feedback was not manipulated experimentally in the present study, in the DIVA model auditory feedback has an important function in the development and maintenance of motor plans necessary for speech production [8]. As one source of evidence for this role, studies inducing changes in auditory feedback showed increased activation bilaterally in the superior temporal gyri [8, 43]. The increased auditory activation observed during the simulated disfluency task in the current study might therefore result from the increased importance of auditory feedback when producing overt speech in a new, non-habitual manner. Alternatively, the increased activation seen in the left-sided auditory cortex may reflect at least in part an increase in auditory input associated with the longer duration of speech production in the non-habitual speech condition. Future studies specifically designed to tease apart the effects of increased duration, repeated delivery of fast temporal information (i.e., repeated consonants at word onset) and increased reliance on feedback on auditory processing are necessary to allow a more in-depth interpretation of the superior temporal cortex results.

Increased activation in the dorsolateral premotor cortex as well as in the inferior parietal cortex suggests that the production of simulated sound repetitions, compared to habitual speech production, required additional resources involved in cognitive manipulations necessary to integrate somatosensory feedback with other information in the speech production system [44]. Furthermore, the parietal cortex is known to be connected to the SMA and dorsal premotor cortex through the superior longitudinal fasciculus (SLF I), which contributes to the regulation of higher aspects of motor behavior [45, 46]. Interestingly, the parietal cortex also plays a role in speech motor programming, with lesions resulting in apraxia of speech [47]. Increased activation in the left parietal cortex has previously been found during both overt and covert comparisons of multisyllabic and monosyllabic words [25]. As the participants were required to utter a longer utterance in the simulated disfluency condition, a length effect may have contributed to increased demands on the speech motor programming. These findings suggest that the production of simulated disfluencies places higher demands not only on systems that are purely related to primary motor and auditory processes in speech production but also on areas important for sensorimotor integration and programming of new speech motor sequences. It would be interesting if future studies could tease the complex interactions between different conditions and stimulus effects on neural activity patterns further apart.

Overall, these findings show that asking participants to speak in a non-habitual manner by simulating disfluencies resulted in increased activation in the neural network of speech production. While no such differences between simulating disfluencies and habitual speech production were identified in a previous study in fluent speakers [19], the observed changes in brain activation in the current study are in line with those provided by other studies on speech modifications. Marchina et al. [3] reported activation changes in the sensorimotor cortex with speech rate changes, with a linear relation between brain activation and speech rate changes only identified in the auditory cortex. This has been interpreted to reflect a direct link between sensory feedback and task demands of non-habitual speech production. The linear relationship between self-paced syllable repetition rates and increased activation in the SMA, left anterior insula, bilateral thalamus, bilateral sensorimotor cortex and cerebellum in Riecker et al. [11]’s study also provides further evidence for the role of this network in non-habitual speech production. This suggests a shift from the use of more efficient ‘automated’ to more elaborate ‘novel production’ networks when people are asked to produce speech in a non-habitual manner.

### Simulated disfluencies versus stuttering

While the deliberate production of sound repetitions by fluent speakers in our study clearly was a simplification of the complex phenomenon of stuttered speech production in PWS, the activation patterns during the production of simulated disfluency in fluent participants showed interesting parallels to those associated with habitual speech production in PWS compared to controls [12–14, 20]. This finding suggests that the increased activation found in the pre- and primary motor cortices, SMA and cerebellum in PWS may reflect, at least in part, differences in the level of automaticity, attention and effort required for speech production, as has previously been suggested by De Nil et al. [19].

In contrast, the increased activation in the auditory cortex in the present study with fluent speakers is opposite to the decrease in auditory cortex activation previously observed during habitual speech production in PWS [16, 48, 49]. However, when asked to simulate dysfluencies, PWS did show an increase in activation in the bilateral superior temporal gyri [19]. Studies investigating the effect of fluency-enhancing conditions have also typically found an increase in activation in the superior temporal gyri in both PWS and controls. This effect seen during choral speech, paced speech, automatic speech and singing has typically been associated with an increase in fluency during such speech conditions [16, 17, 48–50]. However, this interpretation cannot explain the presence of similar increases in auditory cortex activation in fluent control participants [16, 17, 48–50]. Rather than being associated with an increase in speech fluency, it is possible that the increases in fMRI BOLD signal reported during fluency-enhancing conditions reflect the increased need for auditory monitoring when producing speech in a new, non-habitual manner. Thus, some of the activation differences found in the superior temporal gyri in previous studies examining the effects of fluency enhancing speech tasks could have been associated with altered monitoring effort during speech production in addition to other possible influences such as the effect of presenting external auditory pacing cues [17, 51]. In addition, the present results challenge the hypothesis that the absence of significant activation in the superior temporal gyri during habitual speech production in PWS occurs because the repetitions of sounds and syllables in PWS lead to the repeated delivery of the perceptual prediction of the speech sounds to the auditory system, functioning as an inhibitory signal attenuating the activation [13]. In the present study, the introduction of repetitions in the speech did not result in an attenuation of the activity in the superior temporal cortex, but rather an increase in activity in this brain region. This makes it unlikely that the absence of increased activation in the auditory cortices in PWS can be explained as the result of repeatedly receiving the same prediction of auditory input.

### Overt versus covert production

In addition to the effect of the non-habitual simulated disfluency task, a main effect of the mode of speech production was present in the current study. During overt habitual speech production compared to the baseline (see Fig 3 and S3 Table), largely the same pattern of activation was found as in the combined habitual speech compared to baseline contrast (see Fig 1 and S1 Table). During the covert habitual speech task, however, activation was more restricted (see Fig 2 and S2 Table). A direct comparison between the overt and covert speech task revealed large differences between both modes of production, overlapping with the well-established ‘minimal network of speech production’ (see Table 4). The increases in bilateral activation are consistent with requirement to control the articulators and process sensory feedback when producing speech in an overt manner, as found in previous studies [1, 25].

As discussed earlier, our findings on non-habitual speech production suggest that producing simulated disfluencies resulted in increased task demands. This is evidenced by an associated increase in medial premotor cortex activation during covert speech production. This increased reliance on the SMA fits precisely with the non-habitual task requirements on sequencing, timing and initiation of speech. The same task during overt speech production also resulted in increased activation in this area, while an additional larger network of motor and sensory areas also showed increased activation. Furthermore, a significant interaction effect between task and mode of production was present in the left auditory cortex. The interaction between the speech task and the covert and overt mode of production highlights two important issues. Firstly, covert speech cannot be used as a substitute for overt speech when investigating neural activation during speech production. Secondly, studies involving overt speech production tasks are needed to gain a comprehensive understanding of the neural basis underlying habitual and non-habitual speech production [1, 25].

### Word versus pseudoword production

While we also hypothesized that pseudoword versus words production would result in increased demands on the speech system, a significant increase in activation was only present in one right-sided occipital cluster during this comparison. Similarly, in a study specifically focusing on orthographic processing, Tagamets and colleagues [52] found more activation in the right-sided occipito-temporal area in their pseudoword versus word contrast, while they did not find a left-sided difference in this same region. Vigneau and colleagues [53], using a region of interest-based approach to study the function of the visual word form area, also found no difference between pseudowords and words in the left hemisphere, while there was a trend for the right homologue of the visual word form area to be recruited more by pseudowords than by words, and a significant increase in right-sided activation in the nearby inferior occipital gyrus region of interest [53]. However, our study did not identify the additional differences in activation in the left frontal operculum and right cerebellum as shown in a previous study on pseudoword reading [54]. As that study used a covert habitual pseudoword reading task, it is possible that any effect of a further increase in effort due to pseudoword reading in our study was masked by the high level of additional activation already required to overtly produce speech and simulate disfluencies during word reading.

### Limitations

While the sample size of the current study is consistent with that of previous studies on this topic [3, 11], the small sample size is a clear limitation of this study. Replication of our findings in future studies is needed, as is the collection of behavioral data during the MRI acquisition to allow for a more direct comparison of the patterns of brain activation with the behavioral responses in future work.

## Conclusion

The current study aimed to enhance our understanding of the effects of the production of non-habitual speech by asking fluent participants to simulate disfluencies. The results showed that the deliberate production of disfluencies, whether overtly or covertly, resulted in increased brain activation compared to habitual speech production. While increases during covert speech production were restricted to the supplementary motor area, those during overt speech production showed substantial overlap with networks involved in the production of novel motor sequences and of speech. As discussed, these findings have a number of implications for the interpretation of differences in brain functioning identified between people with speech disorders and control speakers, and for the interpretation of the effects of non-habitual speech tasks. An accurate and comprehensive understanding of the neural networks supporting habitual speech production, and those supporting speech modulations, helps to shed light on the effect of such modulations when used in the diagnosis and treatment of speech disorders.

## Supporting information

S1 Table Results on habitual speech compared to baseline. Height threshold of p < 0.001 uncorrected, and cluster-based FWE-corrected p < 0.05 across the whole brain (threshold = 149 voxels). R = right; L = left.
(DOCX)

S2 Table Results of covert habitual speech compared to baseline activation. Height threshold of p < 0.001 uncorrected, and cluster-based FWE-corrected p < 0.05 across the whole brain (threshold = 208 voxels). R = right; L = left.
(DOCX)

S3 Table Results of overt habitual speech compared to baseline activation. Height threshold of p < 0.001 uncorrected, and cluster-based FWE-corrected p < 0.05 across the whole brain (threshold = 138 voxels). R = right; L = left.
(DOCX)

## References

[1] Bohland JW, Guenther FH. An fMRI investigation of syllable sequence production. Neuroimage. 2006;32:821–41. 10.1016/j.neuroimage.2006.04.173 16730195

[2] Golfinopoulos E, Tourville JA, Bohland JW, Ghosh SS, Nieto-Castanon A, Guenther FH. fMRI investigation of unexpected somatosensory feedback perturbation during speech. Neuroimage. 2011;55:1324–38. 10.1016/j.neuroimage.2010.12.065 21195191 PMC3065208

[3] Marchina S, Norton A, Kumar S, Schlaug G. The effect of speech repetition rate on neural activation in healthy adults: Implications for treatment of aphasia and other fluency disorders. Front Hum Neurosci. 2018;12:69 10.3389/fnhum.2018.00069 29535619 PMC5835070

[4] Rong F, Lisette Isenberg A, Sun E, Hickok G. The neuroanatomy of speech sequencing at the syllable level. PLoS ONE. 2018;13(10):e0196381 10.1371/journal.pone.0196381 30300341 PMC6177116

[5] Tremblay P, Deschamps I, Gracco VL. Regional heterogeneity in the processing and the production of speech in the human planum temporale. Cortex. 2013;49:143–57. 10.1016/j.cortex.2011.09.004 22019203

[6] Guenther FH, Ghosh SS, Tourville JA. Neural modeling and imaging of the cortical interactions underlying syllable production. Brain Lang. 2006;96:280–301. 10.1016/j.bandl.2005.06.001 16040108 PMC1473986

[7] Kearney E, Guenther FH. Articulating: The neural mechanisms of speech production. Lang Cogn Neurosci. 2019:Forthcoming. 10.1080/23273798.2019.1589541 31777753 PMC6880942

[8] Tourville JA, Reilly KJ, Guenther FH. Neural mechanisms underlying auditory feedback control of speech. Neuroimage. 2008;39:1429–43. 10.1016/j.neuroimage.2007.09.054 18035557 PMC3658624

[9] Hickok G, Houde J, Rong F. Sensorimotor integration in speech processing: Computational basis and neural organization. Neuron. 2011;69:407–22. 10.1016/j.neuron.2011.01.019 21315253 PMC3057382

[10] Blomgren M. Behavioral treatments for children and adults who stutter: A review. Psychol Res Behav Manag. 2013;2013:9–19. 10.2147/PRBM.S31450 23785248 PMC3682852

[11] Riecker A, Kassubek J, Gröschel K, Grodd W, Ackermann H. The cerebral control of speech tempo: Opposite relationship between speaking rate and BOLD signal changes at striatal and cerebellar structures. Neuroimage. 2006;29:46–53. 10.1016/j.neuroimage.2005.03.046 16085428

[12] Belyk M, Kraft SJ, Brown S. Stuttering as a trait or state–an ALE meta‐analysis of neuroimaging studies. Eur J Neurosci. 2015;41:275–84. 10.1111/ejn.12765 25350867 PMC13140564

[13] Brown S, Ingham RJ, Ingham JC, Laird AR, Fox PT. Stuttered and fluent speech production: An ALE meta-analysis of functional neuroimaging studies. Hum Brain Mapp. 2005;25:105–17. 10.1002/hbm.20140 15846815 PMC6871755

[14] Budde KS, Barron DS, Fox PT. Stuttering, induced fluency, and natural fluency: A hierarchical series of activation likelihood estimation meta-analyses. Brain Lang. 2014;139:99–107. 10.1016/j.bandl.2014.10.002 25463820 PMC4405378

[15] Etchell AC, Civier O, Ballard KJ, Sowman PF. A systematic literature review of neuroimaging research on developmental stuttering between 1995 and 2016. J Fluency Disord. 2018;55:6–45. 10.1016/j.jfludis.2017.03.007 28778745

[16] Fox PTI R. J.; Ingham J. C.; Hirsch T. B.; Downs J. H.; Martin C.; Jerabek P.; Glass T.; Lancaster J. L. A PET study of the neural system of stuttering. Nature. 1996;382:158–62. 10.1038/382158a0 8700204

[17] Toyomura A, Fujii T, Kuriki S. Effect of external auditory pacing on the neural activity of stuttering speakers. Neuroimage. 2011;57:1507–16. 10.1016/j.neuroimage.2011.05.039 21624474

[18] Watkins KE, Smith SM, Davis S, Howell P. Structural and functional abnormalities of the motor system in developmental stuttering. Brain. 2008;131:50–9. 10.1093/brain/awm241 17928317 PMC2492392

[19] De Nil LF, Beal DS, Lafaille SJ, Kroll RM, Crawley AP, Gracco VL. The effects of simulated stuttering and prolonged speech on the neural activation patterns of stuttering and nonstuttering adults. Brain Lang. 2008;107:114–23. 10.1016/j.bandl.2008.07.003 18822455

[20] BelykM, KraftSJ, BrownS. Stuttering as a trait or a state revisited: Motor system involvement in persistent developmental stuttering. Eur J Neurosci. 2017;45:622–4. 10.1111/ejn.13512 28191730

[21] Hickok G, Poeppel D. The cortical organization of speech processing. Nat Rev Neurosci. 2007;8:393–402. 10.1038/nrn2113 17431404

[22] Packman AO M.; Coombes T.; Goodwin A. Stuttering and lexical retrieval. Clin Linguist Phon. 2001;15:487–98.

[23] Namasivayam AK, van Lieshout P. Investigating speech motor practice and learning in people who stutter. J Fluency Disord. 2008;33:32–51. 10.1016/j.jfludis.2007.11.005 18280868

[24] Smith A, Sadagopan N, Walsh B, Weber-Fox C. Increasing phonological complexity reveals heightened instability in inter-articulatory coordination in adults who stutter. J Fluency Disord. 2010;35:1–18. 10.1016/j.jfludis.2009.12.001 20412979 PMC2859203

[25] Shuster LI, Lemieux SK. An fMRI investigation of covertly and overtly produced mono- and multisyllabic words. Brain Lang. 2005;93:20–31. 10.1016/j.bandl.2004.07.007 15766765

[26] Oldfield RC. The assessment and analysis of handedness: The Edinburgh inventory. Neuropsychologia. 1971;9:97–113. 10.1016/0028-3932(71)90067-4 5146491

[27] Van Strien JW. Classificatie van links- en rechtshandige proefpersonen [Classification of left-handed and right-handed subjects]. Ned Tijdschr Psychol. 1992;47:88–92.

[28] Uit den Boogaart PC. Woordfrequenties in geschreven en gesproken Nederlands [Word frequencies in written and spoken Dutch]. Utrecht: Oosthoek, Scheltema & Holkema; 1975.

[29] Ashburner JF, K.J. Unified segmentation. Neuroimage. 2005;26(3):839–51. 10.1016/j.neuroimage.2005.02.018 15955494

[30] Friston KJ, Ashburner JT, Kiebel SJ, Nichols TE, Penny WD, editors. Statistical parametric mapping: The analysis of functional brain images. London: Academic Press; 2007.

[31] McCandliss BD, Cohen L, Dehaene S. The visual word form area: Expertise for reading in the fusiform gyrus. Trends Cogn Sci. 2003;7:293–9. 10.1016/s1364-6613(03)00134-7 12860187

[32] Sharoh DvM, T; Bains L J; Segaert K; Weber K; Hagoort P; Norris D G. Laminar specific fMRI reveals directed interactions in distributed networks during language processing. Proc Natl Acad Sci U S A. 2019;Epub ahead of print. 10.1073/pnas.1907858116 PMC6800353 31570628

[33] Ackermann H, Hertrich I, Ziegler W, Bitzer M, Bien S. Acquired dysfluencies following infarction of the left mesiofrontal cortex. Aphasiology. 1996;10:409–17. 10.1080/02687039608248420

[34] Hertrich I, Dietrich S, Ackermann H. The role of the supplementary motor area for speech and language processing. Neurosci Biobehav Rev. 2016;68:602–10. 10.1016/j.neubiorev.2016.06.030 27343998

[35] Brendel B, Hertrich I, Erb M, Lindner A, Riecker A, Grodd W, et al The contribution of mesiofrontal cortex to the preparation and execution of repetitive syllable productions: An fMRI study. Neuroimage. 2010;50:1219–30. 10.1016/j.neuroimage.2010.01.039 20080191

[36] Shima K, Tanji J. Both supplementary and presupplementary motor areas are crucial for the temporal organization of multiple movements. J Neurophysiol. 1998;80:3247–60. 10.1152/jn.1998.80.6.3247 9862919

[37] Hartwigsen G, Saur D, Price CJ, Baumgaertner A, Ulmer S, Siebner HR. Increased facilitatory connectivity from the pre-sma to the left dorsal premotor cortex during pseudoword repetition. J Cogn Neurosci. 2013;25:580–94. 10.1162/jocn_a_00342 23249347

[38] Ghilardi M-F, Ghez C, Dhawan V, Moeller J, Mentis M, Nakamura T, et al Patterns of regional brain activation associated with different forms of motor learning. Brain Res. 2000;871:127–45. 10.1016/s0006-8993(00)02365-9 10882792

[39] Grafton ST, Hazeltine E, Ivry RB. Motor sequence learning with the nondominant left hand: A PET functional imaging study. Exp Brain Res. 2002;146:369–78. 10.1007/s00221-002-1181-y 12232693

[40] Penhune VB, Steele CJ. Parallel contributions of cerebellar, striatal and M1 mechanisms to motor sequence learning. Behav Brain Res. 2012;226:579–91. 10.1016/j.bbr.2011.09.044 22004979

[41] Segawa JA, Tourville JA, Beal DS, Guenther FH. The neural correlates of speech motor sequence learning. J Cogn Neurosci. 2015;27:819–31. 10.1162/jocn_a_00737 25313656 PMC4344924

[42] Shimizu RE, Wu AD, Knowlton BJ. Cerebellar activation during motor sequence learning is associated with subsequent transfer to new sequences. Behav Neurosci. 2016;130:572–84. 10.1037/bne0000164 27748617

[43] Christoffels IK, Formisano E, Schiller NO. Neural correlates of verbal feedback processing: An fMRI study employing overt speech. Hum Brain Mapp. 2007;28:868–79. 10.1002/hbm.20315 17266104 PMC6871445

[44] Abe M, Hanakawa T. Functional coupling underlying motor and cognitive functions of the dorsal premotor cortex. Behav Brain Res. 2009;198:13–23. 10.1016/j.bbr.2008.10.046 19061921

[45] Jang SH, Hong JH. The anatomical characteristics of superior longitudinal fasciculus I in human brain: Diffusion tensor tractography study. Neurosci Lett. 2012;506:146–8. 10.1016/j.neulet.2011.10.069 22085696

[46] Makris N, Kennedy DN, McInerney S, Sorensen AG, Wang R, Caviness VS Jr, et al Segmentation of subcomponents within the superior longitudinal fascicle in humans: A quantitative, in vivo, DT-MRI study. Cereb Cortex. 2005;15:854–69. 10.1093/cercor/bhh186 15590909

[47] Hilary Ogar JS; Nina Dronkers; Serena Amici; Maria Luisa Gorno-Tempini. Apraxia of speech: an overview. Neurocase. 2005;11:427–32. 10.1080/13554790500263529 16393756

[48] Braun AR, Varga M, Stager S, Schulz G, Selbie S, Maisog JM, et al Altered patterns of cerebral activity during speech and language production in developmental stuttering: An H2 15O positron emission tomography study. Brain. 1997;120:761–84. 10.1093/brain/120.5.761 9183248

[49] Ingham RJ, Fox PT, Costello Ingham J, Zamarripa F. Is overt stuttered speech a prerequisite for the neural activations associated with chronic developmental stuttering? Brain Lang. 2000;75:163–94. 10.1006/brln.2000.2351 11049665

[50] Stager SV, Jeffries KJ, Braun AR. Common features of fluency-evoking conditions studied in stuttering subjects and controls: An H215O PET study. J Fluency Disord. 2003;28:319–36. 10.1016/j.jfludis.2003.08.004 14643068

[51] Neumann K, Euler HA. Neuroimaging in stuttering In: Guitar B, editor. Treatment of stuttering: Established and emerging interventions. Baltimore, MD: Lippincott, Williams & Wilkins; 2009 p. 355–402.

[52] Tagamets MAN J. M.; Chalmers M. L.; Friedman R. B. A parametric approach to orthographic processing in the brain: an fMRI study. J Cogn Neurosci. 2000;12(2):281–97. 10.1162/089892900562101 10771412

[53] Vigneau MJ, G; Mazoyer B; Tzourio-Mazoyer N. Word and non-word reading: what role for the Visual Word Form Area? Neuroimage. 2005;27(3):694–705. 10.1016/j.neuroimage.2005.04.038 15961322

[54] Mechelli A, Gorno-Tempini ML, Price CJ. Neuroimaging studies of word and pseudoword reading: Consistencies, inconsistencies, and limitations. J Cogn Neurosci. 2003;15:260–71. 10.1162/089892903321208196 12676063

