# Supplementary material for: Brain activation during non-habitual speech production: Revisiting the effects of simulated disfluencies in fluent speakers
Source: PLoS One. 2020 Jan 31;15(1):e0228452. doi: 10.1371/journal.pone.0228452 (PMC6993970; doi:10.1371/journal.pone.0228452)
Supplement: S1 Table — Height threshold of p < 0.001 uncorrected, and cluster-based FWE-corrected p < 0.05 across the whole brain (threshold = 149 voxels). R = right; L = left. (DOCX) [file pone.0228452.s001.docx]

**S1 Table. Results on habitual speech compared to baseline.** Height threshold of p < 0.001 uncorrected, and cluster-based FWE-corrected p < 0.05 across the whole brain (threshold = 149 voxels). R = right; L = left.

| **Anatomical region** | **Cluster** | | **Peak** | **MNI coordinates** | | |
| --- | --- | --- | --- | --- | --- | --- |
|  | **FWE-corrected p-value** | **voxel extent** | **t-value** | **x** | **y** | **z** |
| **R Postcentral gyrus** | < 0.001 | 2791 | 13.6 | 56 | -10 | 35 |
| **R Postcentral gyrus** |  |  | 13.1 | 64 | -2 | 25 |
| **R Postcentral gyrus** |  |  | 12.48 | 48 | -12 | 35 |
| **L Postcentral gyrus** | < 0.001 | 4028 | 12.31 | -55 | -8 | 29 |
| **L Postcentral gyrus** |  |  | 11.23 | -49 | -14 | 39 |
| **L Superior temporal gyrus** |  |  | 10.40 | -61 | -6 | 5 |
| **R Cerebellum** | < 0.001 | 2162 | 11.04 | 16 | -62 | -20 |
| **R Cerebellum** |  |  | 10.65 | 26 | -60 | -26 |
| **L Cerebellum** |  |  | 9.86 | -15 | -60 | -20 |
| **L Supplementary motor area** | < 0.001 | 579 | 8.27 | -3 | 3 | 61 |
| **R Supplementary motor area** |  |  | 6.00 | 6 | 5 | 63 |
| **L Supplementary motor area** |  |  | -4.72 | -3 | 11 | 47 |
| **L Amygdala** | 0.004 | 237 | 8.14 | -25 | 1 | -12 |
| **L Hippocampus** |  |  | 8.07 | -15 | -6 | -14 |
| **L Olfactory cortex** |  |  | 3.92 | -15 | 9 | -18 |
| **L Hippocampus** | 0.006 | 221 | 6.36 | -15 | -38 | 9 |
| **L Anterior cingulate gyrus** |  |  | 6.10 | -1 | -30 | 1 |
| **L Cerebellum** |  |  | 5.10 | -9 | -38 | -2 |
| **L Fusiform gyrus** | 0.003 | 247 | 6.00 | -47 | -54 | -20 |
| **L Fusiform gyrus** |  |  | 5.14 | -43 | -46 | -20 |
| **L Inferior temporal gyrus** |  |  | 4.11 | -41 | -40 | -14 |
| **L Inferior frontal gyrus, pars triangularis** | 0.016 | 179 | 5.34 | -39 | 27 | 1 |
| **L Hippocampus** | 0.035 | 149 | 5.03 | -19 | -16 | -14 |
| **L Hippocampus** |  |  | 4.71 | -29 | -16 | -14 |
| **Undefined** | 0.019 | 174 | 4.94 | -1 | 1 | 5 |
| **L Thalamus** |  |  | 4.30 | -13 | -6 | 11 |
| **R Thalamus** |  |  | 4.22 | 4 | -2 | -6 |
