# Supplementary material for: Brain activation during non-habitual speech production: Revisiting the effects of simulated disfluencies in fluent speakers
Source: PLoS One. 2020 Jan 31;15(1):e0228452. doi: 10.1371/journal.pone.0228452 (PMC6993970; doi:10.1371/journal.pone.0228452)
Supplement: S2 Table — Height threshold of p < 0.001 uncorrected, and cluster-based FWE-corrected p < 0.05 across the whole brain (threshold = 208 voxels). R = right; L = left. (DOCX) [file pone.0228452.s002.docx]

**S2 Table. Results of covert habitual speech compared to baseline activation.** Height threshold of p < 0.001 uncorrected, and cluster-based FWE-corrected p < 0.05 across the whole brain (threshold = 208 voxels). R = right; L = left.

| **Anatomical region** | **Cluster** | | **Peak** | **MNI coordinates** | | |
| --- | --- | --- | --- | --- | --- | --- |
|  | **FWE-corrected p-value** | **voxel extent** | **t-value** | **x** | **y** | **z** |
| **L Supplementary motor area** | < 0.001 | 926 | 8.85 | -5 | 3 | 61 |
| **L Supplementary motor area** |  |  | 6.86 | -3 | 11 | 47 |
| **R Supplementary motor area** |  |  | 5.60 | 6 | 7 | 61 |
| **L Postcentral gyrus** | < 0.001 | 1819 | 6.96 | -51 | -6 | 49 |
| **L Postcentral gyrus** |  |  | 6.41 | -51 | -2 | 41 |
| **L Superior temporal pole** |  |  | 6.05 | -57 | 9 | 1 |
| **L Superior temporal gyrus** | < 0.001 | 504 | 6.55 | -55 | -40 | 23 |
| **L Middle temporal gyrus** |  |  | 5.64 | -61 | -28 | 1 |
| **L Middle temporal gyrus** |  |  | 5.06 | -61 | -46 | 7 |
| **R Cerebellum** | < 0.001 | 513 | 5.98 | 26 | -62 | -26 |
| **R Cerebellum** |  |  | 4.81 | 8 | -72 | -18 |
| **R Cerebellum** |  |  | 4.56 | 30 | -56 | -30 |
| **L Fusiform gyrus** | 0.007 | 214 | 5.81 | -43 | -46 | -24 |
| **L Cerebellum** |  |  | 4.84 | -37 | -42 | -28 |
| **R Inferior frontal lobe, pars triangularis** | 0.008 | 208 | 5.47 | 52 | 27 | 31 |
| **R Middle frontal gyrus** |  |  | 5.37 | 46 | 33 | 31 |
