# Supplementary material for: Brain activation during non-habitual speech production: Revisiting the effects of simulated disfluencies in fluent speakers
Source: PLoS One. 2020 Jan 31;15(1):e0228452. doi: 10.1371/journal.pone.0228452 (PMC6993970; doi:10.1371/journal.pone.0228452)
Supplement: S3 Table — Height threshold of p < 0.001 uncorrected, and cluster-based FWE-corrected p < 0.05 across the whole brain (threshold = 138 voxels). R = right; L = left. (DOCX) [file pone.0228452.s003.docx]

**S3 Table. Results of overt habitual speech compared to baseline activation.** Height threshold of p < 0.001 uncorrected, and cluster-based FWE-corrected p < 0.05 across the whole brain (threshold = 138 voxels). R = right; L = left.

| **Anatomical region** | **Cluster** | | **Peak** | **MNI coordinates** | | |
| --- | --- | --- | --- | --- | --- | --- |
|  | **FWE-corrected p-value** | **voxel extent** | **t-value** | **x** | **y** | **z** |
| **R Postcentral gyrus** | < 0.001 | 2635 | 14.20 | 56 | -10 | 35 |
| **R Postcentral gyrus** |  |  | 13.14 | 62 | -2 | 25 |
| **R Postcentral gyrus** |  |  | 12.86 | 48 | -12 | 35 |
| **L Postcentral gyrus** | < 0.001 | 2862 | 13.02 | -55 | -10 | 29 |
| **L Postcentral gyrus** |  |  | 10.70 | -49 | -14 | 39 |
| **L Superior temporal gyrus** |  |  | 9.04 | -61 | -6 | 5 |
| **R Cerebellum** | < 0.001 | 1808 | 10.68 | 16 | -62 | -20 |
| **L Cerebellum** |  |  | 9.54 | -15 | -62 | -20 |
| **R Cerebellum** |  |  | 9.04 | 26 | -60 | -26 |
| **L Amygdala** | 0.009 | 204 | 7.36 | -25 | 1 | -12 |
| **L Hippocampus** |  |  | 7.36 | -15 | -6 | -14 |
| **R Amygdala** | 0.046 | 138 | 6.37 | 28 | 1 | -12 |
| **R Hippocampus** |  |  | 4.67 | 14 | -8 | -14 |
| **R Hippocampus** |  |  | 4.63 | 24 | -12 | -10 |
| **L Brainstem/Thalamus** | 0.036 | 148 | 5.65 | -1 | -30 | 1 |
| **L Thalamus** |  |  | 4.14 | -11 | -22 | 3 |
| **L Supplementary motor area** | 0.015 | 184 | 4.95 | -3 | 1 | 67 |
| **R Supplementary motor area** |  |  | 3.59 | 8 | -6 | 67 |
| **L Thalamus** | 0.031 | 154 | 4.73 | -5 | -4 | 5 |
| **L Thalamus** |  |  | 4.66 | -13 | -6 | 11 |
| **R Thalamus** |  |  | 4.58 | 6 | -4 | 3 |
| **L Putamen** | 0.009 | 206 | 4.68 | -29 | -18 | -4 |
| **L Hippocampus** |  |  | 4.64 | -19 | -24 | -14 |
| **L Hippocampus** |  |  | 4.32 | -21 | -16 | -12 |
